# Tuning pathological brain oscillations with neurofeedback: a systems neuroscience framework

**DOI:** 10.3389/fnhum.2014.01008

**Published:** 2014-12-18

**Authors:** Tomas Ros, Bernard J. Baars, Ruth A. Lanius, Patrik Vuilleumier

**Affiliations:** ^1^Laboratory for Neurology and Imaging of Cognition, Department of Neurosciences, University of GenevaGeneva, Switzerland; ^2^Theoretical Neurobiology, The Neurosciences InstituteLa Jolla, CA, USA; ^3^Department of Psychiatry, University of Western OntarioLondon, ON, Canada

**Keywords:** neurofeedback, brain computer interface (BCI), electroencephalography (EEG), magnetoencephalography (MEG), brain plasticity, brain disorders, neuromodulation, criticality

## Abstract

Neurofeedback (NFB) is emerging as a promising technique that enables self-regulation of ongoing brain oscillations. However, despite a rise in empirical evidence attesting to its clinical benefits, a solid theoretical basis is still lacking on the manner in which NFB is able to achieve these outcomes. The present work attempts to bring together various concepts from neurobiology, engineering, and dynamical systems so as to propose a contemporary theoretical framework for the mechanistic effects of NFB. The objective is to provide a firmly neurophysiological account of NFB, which goes beyond traditional *behaviorist* interpretations that attempt to explain psychological processes solely from a descriptive standpoint whilst treating the brain as a “black box”. To this end, we interlink evidence from experimental findings that encompass a broad range of intrinsic brain phenomena: starting from “bottom-up” mechanisms of neural synchronization, followed by “top-down” regulation of internal brain states, moving to dynamical systems plus control-theoretic principles, and concluding with activity-dependent as well as homeostatic forms of brain plasticity. In support of our framework, we examine the effects of NFB in several brain disorders, including attention-deficit hyperactivity (ADHD) and post-traumatic stress disorder (PTSD). In sum, it is argued that pathological oscillations emerge from an abnormal formation of brain-state attractor landscape(s). The central thesis put forward is that NFB tunes brain oscillations toward a homeostatic set-point which affords an optimal balance between network flexibility and stability (i.e., self-organised criticality (SOC)).

*“While we can conceive of a sum being composed gradually, a system as total of parts…has to be conceived of as being composed instantly” –* Von Bertalanffy, General System Theory (1969)

## (De)synchronized brain states

In 1934, a few years after the initial discovery of the electroencephalogram (EEG) by Hans Berger, the British magazine Spectator reported on a remarkable public demonstration (Walter, 1934, p. 479):

“Adrian and Matthews recently gave an elegant demonstration of these cortical potentials. […] when the subject’s eyes were open the line was irregular, but when his eyes were shut it showed a regular series of large waves occurring at about ten a second. […] then came the surprise. When the subject shut his eyes and was given a simple problem in mental arithmetic, as long as he was working it out the waves were absent and the line was irregular, as when his eyes were open. When he had solved the problem, the waves reappeared. […] so, with this technique, thought would seem to be a negative sort of thing: a breaking of the synchronized activity of enormous numbers of cells into an individualized working.”

**Figure 1:**
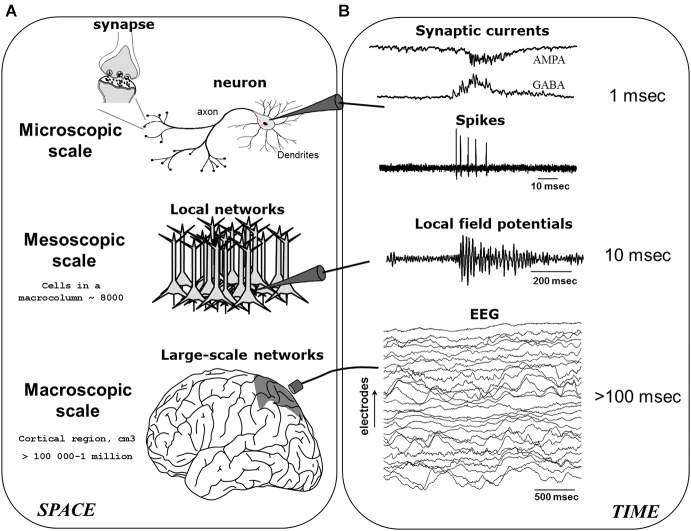
**The generation of electroencephalogram (EEG) network oscillations**. EEG signals are generated by the integration of neural activity at multiple spatial **(A)** and temporal **(B)** scales. After Le Van Quyen (2011).

A basic ingredient sufficient for producing neuronal oscillations is the mutual coupling between excitatory (E) and inhibitory (I) neurons (Wang, 2010). Here, as the E-neurons fire they activate the I-neurons, which after some delay retroactively silence the E-neurons, and so *ad perpetuum*. In essence, this E-I connectivity serves to keep neuronal activity within a restricted range, as purely E-E or I-I coupling would risk producing run-away excitation or inhibition (although such connections naturally also exist). This recurrent feedback mechanism, scaled-up to contain an intricate web of millions of excitatory and inhibitory neurons (as well as glia), ultimately contributes to what are commonly known as brain oscillations or “brainwaves” (Buzsáki and Watson, 2012). Brain oscillations may be recorded via invasive or non-invasive electrodes, given that neuronal activity is reflected in the minute fluctuations of electromagnetic field potentials, which are themselves generated by ionic exchanges at the cell-membrane and the synapse during neuronal communication (Nunez, 2000; Buzsáki et al., 2012). As seen in Figure 1, when neuronal activities occur in a spatially circumscribed region and become temporally synchronized, their local field potentials (LFPs) are then strongly summated giving rise to large amplitude electroencephalogram (EEG) or magnetoencephalogram (MEG) rhythms. In what follows, we will mainly focus on the modulation of low-frequency M/EEG oscillations (typically <60 Hz), which represent the largest part of neuroelectric activity generated by the brain and which can be recorded noninvasively. Specifically, studies have established that the *amplitude* of M/EEG oscillations varies primarily as a function of the number, strength and phase-locking (“synchronization”) of cortical synaptic activities (Nunez, 2000).

Hence, metaphorically akin to a “standing-waves” generated by a crowd of spectators, the size (amplitude) of an oscillation is proportional to the degree to which a group of persons (neurons) temporally stay “in sync” (synchronize) with each other. Conversely, reductions in amplitude result from a breakdown of such synchronization, in accordance with the historical expression: *desynchronization*. Likewise, the speed (frequency) of the wave will be determined by how quickly the individual elements rise and decay (Nunez, 2000), and this will depend on the intrinsic nature (resonance) of the person (neuron). Here, a greater (lower) number of oscillations occurring in the same period of time will equate to faster (slower) frequencies. The M/EEG may therefore be considered as an accurate non-invasive indicator of coordinated synaptic activity across cortical networks. In general, the M/EEG frequency spectrum has been traditionally divided into the following bands: infraslow (<1 Hz), delta (1–4 Hz), theta (4–7 Hz), alpha (7–12 Hz), spindle (12–15 Hz), beta (15–30 Hz), and gamma (>30 Hz).

Historically, EEG synchronization patterns were discovered to differentiate levels of psychological arousal in the progression from deep sleep to wakefulness, to high alertness (Jasper and Droogleever-Fortuyn, 1948). Low-frequency delta (1–4 Hz) waves were found to dominate deeper sleep states, while during lighter or more activated (REM) sleep the frequencies are more accelerated, but slower than in waking states. In relaxed wakefulness there was an emergence of the alpha (7–12 Hz) rhythm that gave rise to faster beta (15–30 Hz) and gamma (>30 Hz) frequencies upon activation of cognitive or attentional resources (Steriade et al., 1993; Gervasoni et al., 2004). In parallel to this acceleration of frequencies during arousal, there was also a more desynchronized or “activated” tracing of reduced amplitudes (as reported by Walter above). With the discovery that the ascending reticular activating system (ARAS; Moruzzi and Magoun, 1949) was responsible for consciousness and the sleep-wake cycle, some of the most important findings were that lesions in the ARAS abolished the aforementioned “activation” of the EEG whilst increasing episodes of sleep and motor inactivity (Lindsley et al., 1950). Interestingly, progressively greater degrees of EEG activation could be provoked by simple electrical stimulation of the brainstem (Moruzzi and Magoun, 1949), enhancing the precision and speed of visual discrimination in monkeys (Fuster, 1958). Consequently, EEG activation is widely regarded to be necessary for the emergence as well as the characteristic nature of consciousness (Villablanca, 2004), which once established, invites a fascinating question: how is intrinsic brain activity regulated further to give rise to volitional control of cognition? Here, synchronization patterns of neural activity suggest distinct “intrinsic states” that are modulated endogenously (e.g., via neuromodulation, plasticity), independently of external influences (e.g., sensory, pharmacological or electromagnetic stimuli). This has been unequivocally demonstrated by Poulet and Petersen who, upon severing rats’ sensory pathways, showed that internal state transitions during active vs. quiet behavior were uniquely reflected in cortical (de)synchronization patterns (Poulet and Petersen, 2008). On the other hand, a large body of evidence in humans points to the key role of cortical oscillations in top-down processing during attention and cognition (Palva and Palva, 2012). Thus, during waking consciousness, there is a critical involvement of higher-order cortical regions in orchestrating the phasic (i.e., sub-second) shifts between intrinsic brain states, either *cortico-cortically* or *cortico-subcortically* (Harris and Thiele, 2011). A good example of the former is the way motor cortex is able to concurrently trigger desynchronization of somatosensory cortex (Zagha et al., 2013). Similarly, there is evidence of a direct cortico-subcortical dialog during maintenance of wakefulness (in a novel environment), since destruction of either anterior cingulate cortex or locus coeruleus is sufficient to block exploratory activity and associated EEG activation (Gompf et al., 2010). Moreover, when major anatomical routes are severed, as with targeted lesions to the lateral prefrontal cortex plus corpus callosum, it leads to increased distractibility coupled with abnormally high neural synchronization in visual areas during attention (Gregoriou et al., 2014).

**Figure 2:**
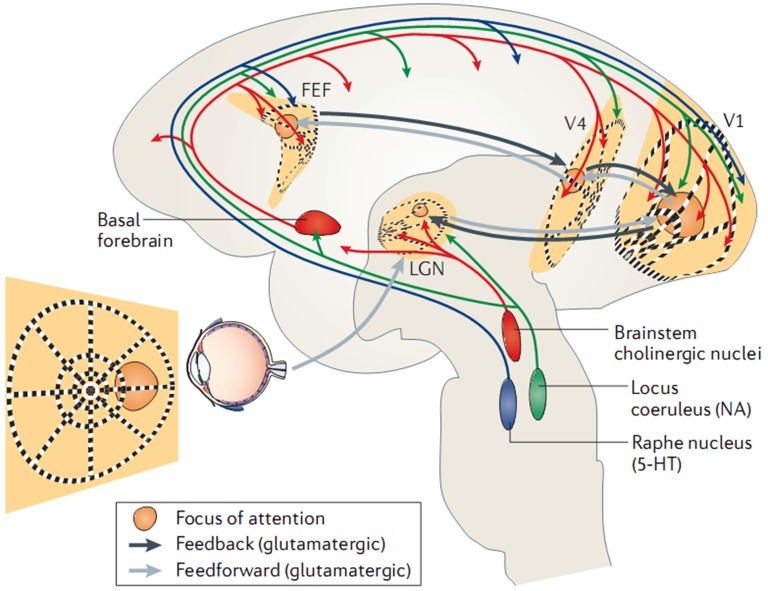
**Control of EEG (de)synchronization via shifts in intrinsic brain state**. Here, a recurrent functional circuit subserving top-down attention is triggered by neocortical structures (black lines; frontal-eye fields (FEF); visual cortices (V1/V4), and reinforced by ascending neuromodulatory pathways (blue/green/red). Adapted with permission from Harris and Thiele (2011).

In parallel and at the molecular level, investigations indicate that tonic and phasic activation of the cortex is dependent on a family of neuromodulators released by the brainstem and/or basal forebrain, including dopamine, acetylcholine, and noradrenaline. It has become evident that both the (tonic) sleep-wake cycle and (phasic) top-down shifts in brain-state are regulated by an intricate interplay of neuromodulators (for a detailed review see Lee and Dan, 2012). Accordingly, attentional behavior and distinct EEG rhythms have been reported to be affected by the lesion and pharmacological blockade of noradrenergic pathways (Delagrange et al., 1993) and enhanced by cholinergic agonists (Bauer et al., 2012). Moreover, local application of acetylcholine in the monkey primary visual cortex is able to enhance the behavioral modulation of neuronal firing rates (Herrero et al., 2008). Such effects have been verified directly *in vitro*, as for example, dopaminergic antagonists are found to increase EEG spectral power (0–20 Hz) while agonists decrease it (Sebban et al., 1999), and this has been specifically linked to activation of dopamine receptors (Chen et al., 2013). Similarly, optogenetic studies report EEG desynchronization following selective activation of cholinergic (Kalmbach and Waters, 2014) or noradrenergic (Carter et al., 2010) neurons. In sum, the studies above reveal that in addition to the tonic sleep-wake cycle, cortical-subcortical neuromodulatory circuits are able to control brain oscillations phasically (i.e., on a sub-second time scale) in a top-down manner, which is illustrated schematically in Figure 2.

However, the observations above invite the inevitable question: what is the functional significance of such synchronized and desynchronized states? Why does the cortex, for example, display highly-synchronous low-frequency states during unconsciousness, and what necessitates the desynchronized, higher-frequency oscillations of wakefulness (Gervasoni et al., 2004)? Neuroscience is of course still answering these questions, and there is no encompassing theory as yet. However, several emerging perspectives are beginning to shed light on these phenomena.

The first perspective involves the observation that upon intracellular recording of corticothalamic (Contreras and Steriade, 1995) as well as corticospinal (Ezure and Oshima, 1981) neurons, cell-membrane depolarization (excitation) is found to be greater during desynchronized EEG states. Conversely, during sleep, membrane potentials are more hyperpolarized (inhibited) leading to slower oscillations which are characterized by large alternating cortical up (higher excitability) and down (lower excitability) states (Castro-Alamancos, 2009). Thus, in the simplest scenario, desynchronization stems from a rise in neuromodulators which elevate (depolarize) membrane potentials and their voltage-gated-ion channels closer to their firing threshold, enhancing their sensitivity to incoming sensory inputs (Castro-Alamancos, 2004; Wang et al., 2014). This is the case, for example, for the dominant low-frequency rhythm of sensory cortex (“alpha” rhythm), where trial-by-trial variations in detection performance (Ergenoglu et al., 2004; Haegens et al., 2011) and attentional state (Fries et al., 2001; Fan et al., 2007; Macdonald et al., 2011) are predicted by greater degrees of desynchronization. Similarly, desynchronized states are reported to sharpen visual receptive fields (Wörgötter et al., 1998) whilst shortening their response latencies (Wang et al., 2014), concomitant with increases in excitability (Romei et al., 2008) and neuronal spike rate (Haegens et al., 2011). In this way, neuronal synchronization may perform functional “gating” of sensory input by opening or closing neuronal excitability windows (Jensen and Mazaheri, 2010; Luczak et al., 2013). The second perspective involves the fact that desynchronized states have been attributed to larger background synaptic activity, which leads to higher resting membrane conductance (Wang et al., 2014). Such high-conductance states result in enhanced neuronal responsiveness, by boosting signal-to-noise ratios via “stochastic resonance” mechanisms (Destexhe, 2007). From yet another perspective, desynchronized patterns may be seen to minimize functional correlations of synaptic activities, thus maximizing their informational complexity (called *entropy*). Several studies report reduced inter-neuronal correlations during attention (Cohen and Maunsell, 2009) and memory formation (Bermudez Contreras et al., 2013) that imply mechanisms of active decorrelation (Ecker et al., 2010; Renart et al., 2010). According to this perspective, states of synchronized/desynchronized low-frequency activity have been proposed to coincide with decreased/increased information content (Hanslmayr et al., 2012). This notion has received direct experimental support during perceptual-decision making (Werkle-Bergner et al., 2014). As a corollary, extremes of too much or too little synchronization would both have negative consequences for population coding, as this would lead to abnormal redundancy of information, reflective of a highly ordered or chaotic system (Hanslmayr et al., 2012), respectively.

In general, the covered evidence suggests that low-frequency oscillations appear to limit the complexity of available computational states, so why should they feature so prominently in the brain? A potential biological compromise may be that oscillations enable segregated communication channels to be established in the brain, which would prevent a disorganized mixing of processing streams. Thus far, we have mainly considered the features of locally synchronized activities (i.e., arising within circumscribed anatomical regions), yet there is equally evidence of long-range synchronization phenomena, spanning distributed regions? Although this complex topic is beyond the scope of this paper, we touch upon it briefly in light of its relevance to pathological states. In essence, distributed brain regions have been observed to functionally co-activate on a variety of measures, including synchronization of phase, frequency, or amplitude (Engel et al., 2013). Recent studies indicate that these mechanisms enable the collective binding of neural assemblies to form functional networks independent of inter-neuron distance (Canolty et al., 2010), governing diverse processes such as attention (Doesburg et al., 2009a), memory retrieval (Foster et al., 2013; Watrous et al., 2013), and learning (Koralek et al., 2013). A putative mechanism by which this occurs involves the well-known “communication through coherence” theory (Fries, 2005), which posits that distributed neuronal assemblies are bound together by alignment of their oscillatory phases (i.e., phase-locking), thus enabling neuronal spiking to be transmitted through temporally-distinct excitability windows (e.g., low/high excitability states would respectively correspond to oscillation peaks/troughs). Mathematical modeling indicates that such inter-neuronal communication channels can become degraded if the sender-receiver populations become “out-of-tune” with each other in amplitude, phase, or frequency (Akam and Kullmann, 2012; Shin and Cho, 2013), echoing the relationship between broadcasting stations and radios. Moreover, it has become evident that such “synchrony” patterns of spontaneous brain activity frequently form well-defined, reproducible topographies across individuals, known as resting-state networks (Chu et al., 2012; Baker et al., 2014). It is now well-established that the intrinsic dynamics of these networks strongly influence “ongoing” processing of stimuli (Mayhew et al., 2013), as well as a wide-range of cognitive-behavioral functions (Sadaghiani and Kleinschmidt, 2013). Hence, it is not difficult to envisage the emergence of a dynamic interplay between local- and network-oscillation states, as the former would influence the latter via long-range connections (Zemankovics et al., 2013; Cabral et al., 2014), and vice versa (Doesburg et al., 2009a; Shin and Cho, 2013). Likewise, depending on behavioral state, distributed neurons may combine to form distinct functional connectivity networks by reorganizing their oscillatory modes (Quilichini and Bernard, 2012), given that neuromodulators released during different behaviors can preferentially activate neural populations by varying their “resonant frequencies” (Tseng et al., 2014). The general purpose of such synchronization patterns is to enable the simultaneous segregation/integration of distributed functional pathways (Varela et al., 2001; Buzsáki and Watson, 2012) in support of adaptive behavior (Krichmar, 2008). As we will see in the next section, adaptive behavior and consciousness can be altered when this delicate oscillatory balance is disturbed.

In summary, this introductory section highlights several important points: (i) neuronal synchronization is regulated by neuromodulators that govern behavioral states; (ii) both neuronal synchronization and behavioral state remain under top-down control during wakefulness; and (iii) neuronal synchronization modulates the excitability and functional segregation/integration of cerebral circuits.

## Normal and pathological oscillations

The notion of *pathological oscillations* is by definition predicated on the existence of “normal” oscillatory activity. Thus, a science of (ab)normal oscillations should also be supported by observations that quantitative measures (e.g., amplitude, frequency, phase-locking) of low-frequency oscillations exhibit a stable and reproducible distribution in neurologically-healthy populations, i.e., occur in a typical physiological range. Accordingly, studies report good reliability of conventional EEG measures in healthy populations within task/resting conditions and across time (Fingelkurts et al., 2006; Gudmundsson et al., 2007; Näpflin et al., 2007, 2008). This is qualified by a proviso that EEG parameters are not static from birth, but follow an established developmental trajectory consisting of a frequency acceleration of the dominant resting rhythm, and a decrease of the overall spectral power until adulthood (Dustman et al., 1999), reputedly due to synaptic pruning (Whitford et al., 2007). Such age-matched measures from healthy reference populations are implicitly used by neuroscience studies that seek to uncover meaningful differences with pathophysiological conditions. The literature on this topic is vast, but we provide a few representative examples of low-frequency EEG abnormalities prevalent in brain disorders. For instance, slower-waves (e.g., theta 4–8 Hz) are reported to be globally elevated in attentional deficit hyperactivity disorder (Clarke et al., 2007) which may in part be mediated by a slowed frequency of the dominant resting (“alpha”) rhythm (Arns et al., 2008). Similarly, obsessive-compulsive disorder (OCD) patients demonstrate low-frequency power excess (2–6 Hz) in the resting state, which appears to be relatively localized to the subgenual anterior cingulate gyrus and adjacent limbic structures (Kopřivová et al., 2011). Another example is post-traumatic stress disorder (PTSD), which is observed to have both decreased power and accelerated frequency of the alpha rhythm, potentially reflecting cortical hyperarousal (Jokić-Begić and Begić, 2003; Wahbeh and Oken, 2013). In contrast, schizophrenia is distinguished by synchronization deficits of faster gamma (>30 Hz) rhythms during active processing (Grützner et al., 2013; Ramyead et al., 2014) that are found to inversely correlate with levels of the inhibitory neurotransmitter GABA (Ramyead et al., 2014). Alzheimer’s patients display a pronounced lack of alpha-rhythms which positively correlates with hippocampal volume (Babiloni et al., 2009). The list is virtually endless given the plethora as well as complexity of disorders, and the interested reader is referred to comprehensive reviews on the subject (Coburn et al., 2006; Uhlhaas and Singer, 2006). Importantly, EEG can also be employed to assess recovery or response to treatment. For example, reduced delta (2–4 Hz) rhythm amplitude can be used as a biomarker of long-term recovery from ischemic cerebral stroke (Cuspineda et al., 2007), positively correlating with perfusion of cortical lesions (Finnigan et al., 2004). Faster beta band hyper-synchronization is related to motor impairment in Parkinson’s patients, and its disappearance is associated with successful treatment with both medication (Silberstein et al., 2005) or deep brain stimulation (DBS; Little and Brown, 2014). Interestingly, administration of psychostimulants improves behavior in attention-deficit hyperactivity disorder (ADHD) and is found to normalize slow-wave patterns of EEG activity (Clarke et al., 2007). However a non-trivial caveat is that the notion of EEG abnormality (and its normalization following treatment) appears to be state-dependent (Arns et al., 2009), meaning that an appropriate behavioral task(s) may be necessary to uncover disorder-specific patterns, thereby evolving on the passive resting-state recording. For example, oscillatory and topographical differences between ADHD and healthy subjects manifest distinctly (or not at all) depending on the attentional task used (Sohn et al., 2010; Buyck and Wiersema, 2014).

**Figure 3:**
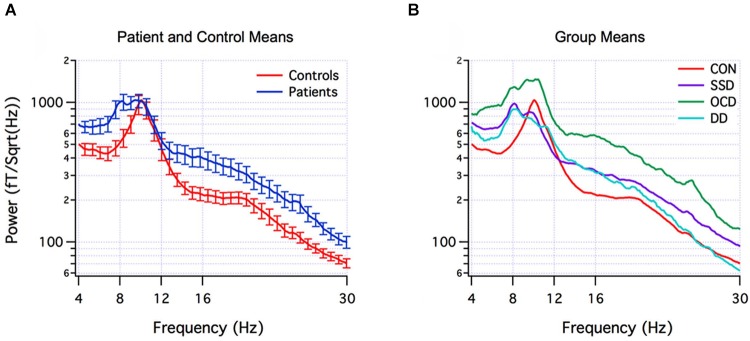
**EEG spectral signatures of healthy and psychiatric populations. (Panel A)** Mean (± SEM) EEG power spectra of healthy control subjects (red) and psychiatric patients (blue). **(Panel B)** Mean subgroup spectra for controls (CON) (red, *n* = 18), schizophrenia spectrum disorder (SSD) (purple, *n* = 14), obsessive-compulsive disorder (OCD) (green, *n* = 10), depression disorder (DD) (light blue, *n* = 5). From Schulman et al. (2011).

The actual neuromolecular processes underpinning aberrant oscillations are likely to be both complex and diverse across pathologies. Nevertheless, a theoretical model termed *thalamocortical dysrhythmia* (TCD) has been put forward to explain the pronounced spectral alterations observed in number of brain disorders (Llinás et al., 2005; Schulman et al., 2011) which are depicted in Figure 3 for psychiatric populations. In addition, several reviews have provided in-depth treatments of the diverse cellular mechanisms that appear to subserve (ab)normal brain oscillations (Steriade et al., 1990; Llinás et al., 2005; Wang, 2010). In this respect, however, a fundamental limitation is that disorders conventionally categorized via cognitive/behavioral dimensions are not necessarily neurobiologically homogenous, i.e., multiple neural subtypes may exist within each disorder called “endophenotypes”. This can be explained by the presence of multiple comorbidities and the possibility for *similar* behavioral patterns to be generated by *dissimilar* neural substrates (Tognoli and Kelso, 2014). Mounting evidence for this is provided by reports of heterogenous EEG profiles within ADHD (Clarke et al., 2001), depression (Pizzagalli et al., 2002), and schizophrenia (John et al., 2007) patient groups, to name a few. A compounding problem is that many studies in the field consist of small sample sizes (*n* < 50) which, upon averaging, may limit their sensitivity for uncovering distinctive subtypes of EEG signatures. Thus, a mixture of heterogeneity and selective sampling could be a feasible explanation for both the similar and contradicting EEG signatures reported between and within disorders, respectively. A complementary but more statistically-powerful method involves developing and utilizing a *normative database*, which enables patient groups, and importantly *single individuals* to be compared to a much larger sampling distribution of the healthy population (typically *n* > 500) (Thatcher and Lubar, 2009). This approach, originally termed “neurometrics”, was first systematically developed by John et al. (1977), by sampling topographical EEG across the full human lifespan and classifying a variety of brain disorders based on their spectral signatures (John et al., 1988). Over time, and upon establishment of several databases (Thatcher and Lubar, 2009), the general approach of examining or classifying patients based on multivariate EEG patterns was re-christened as quantitative EEG (qEEG), to differentiate it from qualitative EEG interpretation. A key objective of qEEG has been to improve sensitivity (i.e., low false-negative) and specificity (i.e., low false-positive) rates in order to aid clinical diagnosis and treatment (Coburn et al., 2006). Recent efforts have concentrated on identifying EEG biomarkers that are recurrently expressed by particular (sub)types of brain disorders (Coburn et al., 2006). Thus for example, in a blinded sample of 159 children and adolescents, an elevated theta/beta power ratio was able to identify ADHD with a remarkable 87% sensitivity and 94% specificity (Snyder et al., 2008); however, this ADHD sample was relatively homogenous, with only 1% of children demonstrating a familiar subtype of increased beta power. It is important to note that biomarker differences can also appear between different age-groups of the same disorder, e.g., ADHD (Poil et al., 2014). Hence the key message is that brain disorders seem to fall on a multi-dimensional continuum, with scarce evidence to support a one-to-one mapping between specific EEG abnormalities and cognitive-behavioral traits (i.e., one cannot be unequivocally inferred from the other). This does not negate the existence of a relationship *per se*, but rather that it is complex and has the interesting property of *degeneracy* (Edelman and Gally, 2001).

## The brain as a dynamical system

In light of the complex linkage between brain activity and behavior, scientists have tried to expand the scope of their analyses by introducing more *dynamical* measures of neuronal oscillations, such as burst (Montez et al., 2009), fractal (Jagadisha et al., 2003), and entropy metrics (Takahashi et al., 2010). The dynamical designation relates to considering the *temporal* evolution of a brain signal, as this can be overlooked upon computing the traditional Fourier transform (e.g., power vs. frequency). In other words, introducing time into analyses takes into account the fact that brain oscillations are non-stationary, i.e., their oscillatory parameters are not constant across time. Interestingly, such time-varying behavior can be accommodated within the framework of *dynamical systems theory*, opening the door to a whole new world of exotic phenomena: bifurcations, attractors, dynamic repertoires, and phase transitions. Although we cannot give these full treatment (for an excellent review see Stam, 2005, a few visual analogies may serve as an introduction. In essence, a system’s operation can be represented in *state-space*, which is best visualized as a multidimensional energy landscape.

**Figure 4:**
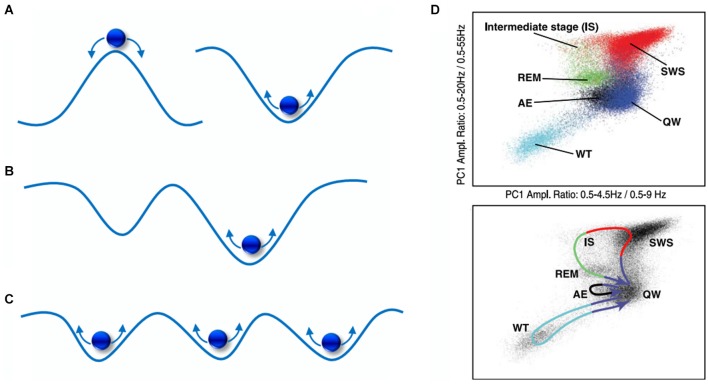
**A visual portrayal of state-space landscapes.** **(A)** A hill and valley representation of a repellor (left) and an attractor (right); **(B)** the shallow attractor (left) has a shorter dwell-time than the deeper attractor (right); **(C)** a multi-attractor landscape exhibiting multistability; **(D)** EEG state transitions during sleep-wake activity in the rat, comprising of whisker twitching (WT), active exploration (AE), quiet wake (QW), rapid-eye movement (REM), slow-wave sleep (SWS), intermediate stage (IS). From Gervasoni et al. (2004).

**Figure 5:**
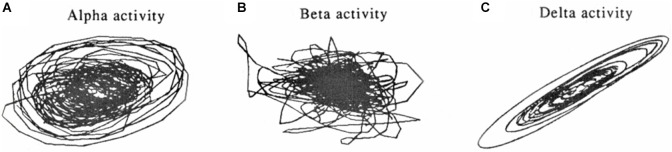
**Phase-space dynamical plots of EEG rhythms during sleep**. Attractor-like (limit cycle) shapes are more pronounced for alpha **(A)** and delta rhythms **(C)**, compared to the beta rhythm **(B)**. From Pradhan et al. (1995).

As depicted in Figure 4A, this can be simplified to 2-dimensions and envisaged as a ball with random energy (i.e., noise) traversing hills and valleys. Here, the ball (dynamic state) will experience greater *stability* (i.e., larger dwell-time) within valleys of low potential, known as *basins of attraction*, and less so at the hills, known as repellors. In Figure 4B, a deeper *attractor* (right) offers more stability than a shallower one (left), as it will keep the ball within its basin at relatively greater energy perturbations. However, is there explicit evidence of attractor-like signatures in the brain? Quite wonderfully, it seems that oscillations with distinct frequency “peaks” exhibit attractor properties, such as delta and alpha rhythms (Pradhan et al., 1995; Freyer et al., 2011; MacIver and Bland, 2014). As illustrated in Figure 5, when common brain rhythms are plotted in their respective phase-space, slower (alpha/delta) rhythms present stronger attractor-related “orbits” than faster ones (beta) (Pradhan et al., 1995). Equally so, the “waxing-and-waning” of alpha oscillations has been observed to follow a *bimodal* distribution, the latter implying that distinct dynamical processes arising from a single cortical region are alternately expressed (Freyer et al., 2009). Put differently, alternating (de)synchronization patterns can be understood to display non-random statistical properties, exemplified by different temporal distributions (i.e., dwell-times) of low vs. high synchronization states. Such state transitions, known as *bifurcations*, may be driven by both internal (Freyer et al., 2011) as well as external (Avella Gonzalez et al., 2012) network activity. Secondly, phasic or tonic alternations *between* EEG frequencies may also be seen as reflecting dynamic transitions between attractors. One of the clearest examples can be found in the sleep-wake cycle which reveals distinct yet recurring *states* as well as *trajectories* corresponding to each neurobehavioral transition as shown in Figure 4D (Gervasoni et al., 2004).

This conveniently brings us the concept of multistability, illustrated in Figure 4C. Here, a ball with a continuous source of energy may revisit multiple states without settling into any of them permanently (e.g., sleep-wake states, sensory percepts, memories, network configurations). Thus, it has been proposed that brain function may also exhibit multistability (Deco and Jirsa, 2012; Tognoli and Kelso, 2014), a property of systems that is neither stable nor totally unstable, but which temporally alternates between multiple, mutually exclusive states referred to as the system’s *dynamic repertoire* (Ghosh et al., 2008). Evidence for recurring, spatiotemporally discrete brain patterns has emerged from both EEG (Van de Ville et al., 2010; Baker et al., 2014; Mehrkanoon et al., 2014) and fMRI (Hellyer et al., 2014; Tagliazucchi et al., 2014) during tasks and resting-states. The tentative implication is that such patterns reflect dynamic circuit motifs which coordinate specific computational operations, including gating and integration of inputs (Womelsdorf et al., 2014) as well as higher-order modular processing subserved by large-scale brain networks (Baker et al., 2014; Hellyer et al., 2014). The direct impact of neural multistability on cognition is beautifully exemplified by the phenomenon of bistable perception (Braun and Mattia, 2010), where perceptual alternations occur in spite of constant sensory stimulation (e.g., Necker Cube, Vase-Faces illusion). Here, a host of EEG parameters are reported to predict perceptual transitions, including alpha and gamma oscillations (Kornmeier and Bach, 2012).

Last but not least, dynamical systems theory points to a related, equally captivating topic: criticality. Derived from laws of thermodynamics, critical systems are said to operate at the *edge of chaos*, that is to say, at an optimal “sweet-spot” between order and disorder, which paradoxically affords flexibility *and* stability (!) (Pastukhov et al., 2013; Hellyer et al., 2014). Practically speaking, the brain exhibits both stability when generating consistent behavior, and variability when learning new patterns. By navigating critical boundaries, complex systems fundamentally avoid being dominated by one of two extreme poles. The first, belonging to the *supercritical* regime reflects highly disordered dynamics typified by very brief dwell-times and unpredictable state transitions, i.e., random noise. The second pole belongs to the *subcritical* regime and is characterized by elements so excessively coupled that they converge on a globally stable state, i.e., absolute order. Respective examples of the former and latter are the behavior of a gas and a simple pendulum. Interestingly, from the oscillatory point of view, computing the power spectral density of a gas gives a uniformly flat spectrum, whereas a pendulum produces a single, well-defined frequency peak. Hence, in the frequency domain, we can respectively glimpse features of a stochastic system without any attractors and that of a harmonic oscillator containing a single attractor (called a limit-cycle). Accordingly, EEG activities appear to be a mixture of high-dimensional noise-driven processes as well as low-dimensional phenomena such as rhythmic limit-cycles (e.g., alpha oscillations) (Stam, 2005; Freyer et al., 2011). But this is insufficient to prove the brain actually operates near criticality. Now, if we were to remove the most prominent oscillatory peaks from the EEG power spectrum, we could then observe its background scaling. This is recognized to have a hyperbolic shape (1/f) known as “pink noise”, curiously poised between “white noise” (flat) and “brown noise” (1/f^2^) spectra, both of which are stochastically generated. And so arose a stunning insight: such 1/f scaling might actually reflect scale-free (i.e., fractal) processes characteristic of self-organized criticality (SOC), an active mechanism that maintains complex systems in a critical state (Bak et al., 1987). Since then, an ever-growing body of work has emerged on neuronal avalanches and temporal auto-correlations suggesting that the brain may indeed operate near criticality (reviewed by Hesse and Gross, 2014), which would endow it with maximal dynamic range, information transmission and capacity (Shew and Plenz, 2013). Importantly, *in vitro* as well as modeling studies suggest that tuning the excitation/inhibition balance (e.g., via neuromodulators) is able to alter such putative measures of criticality (Monto et al., 2007; Poil et al., 2012), can be predictive of behavior (Smit et al., 2013), and has been shown to be abnormal in several brain disorders (e.g., Montez et al., 2009).

Hence, tying all the pieces together, we speculate that abnormal synchronization patterns emerge from plastic changes in brain-state attractor landscape(s), which mutually* shape* and* are shaped by* system criticality, manifesting as subcritical or supercritical regimes that characterize disease (Montez et al., 2009; Poil et al., 2012); and secondly, that restoring the pathological oscillatory signatures toward normative values found in the healthy population (e.g., power, phase-locking, peak frequency, 1/f) would restore in good measure the near-critical regime required for optimal information processing (Thatcher et al., 2009; Shew and Plenz, 2013).

## Neurofeedback: unlocking direct control of brain oscillations

In principle, all that is required to implement neurofeedback (NFB) is an EEG amplifier connected to a computer that provides *real-time* information about a person’s brain activity, otherwise known as brain-computer interface (BCI). In so-called “open-loop” applications, specific oscillatory patterns can be recognized by the computer and used to issue a command, helping participants interact with the environment independent of the body’s conventional mode of output, which is motor. This is the basis of BCI applications that enable quadriplegics to steer a wheelchair (Millan et al., 2009) or “locked-in” patients to communicate (Birbaumer et al., 2006). On the other hand, in a closed-loop or “NFB” design, a sensory representation of the brain activity is fed-back to users continuously in real-time (as a video game for example), with the aim of controlling the activity *in and of itself*. Put more simply, a NFB interface acts as a virtual “mirror” for neuronal oscillations occurring within the brain, empowering a person to explicitly modify them.

The rationale for NFB can be best understood by taking a historical viewpoint “upon the shoulders of giants”. In this case, NFB’s foundations may be nicely summarized by a pair of pivotal discoveries. The first one took place a half-century ago, in the mid-1960s, when Kamiya originally demonstrated that volitional control of human brain oscillations can be achieved with sensory feedback from a BCI (for a historical account, see Kamiya, 2011). In this case real-time information of alpha rhythm activity was provided to users via auditory feedback, who reported mental states of relaxation and “letting go” during higher synchronization levels. This phenomenon, since described as “operant conditioning”, was later shown to be possible in animals (Wyrwicka and Sterman, 1968; Fetz, 1969). In essence, it demonstrated for the first time the feasibility of achieving *real-time control* of brain activity via sensory feedback channels. Shortly after arrived a second seminal discovery: in cats, NFB was observed to induce long-term changes in spontaneous oscillations outside of the training period i.e., during sleep (Sterman et al., 1970). During what may be described as a serendipitous breakthrough, training such (spindle) oscillations was discovered to have a neuroprotective effect against epileptic seizures in cats (Sterman et al., 1969). Hence, this finding revealed for the first time NFB’s ability to induce brain* plasticity*, giving rise to a direct clinical benefit. The union of these two historic discoveries: the feasible control of human EEG rhythms with NFB—on the one hand, and long-term induction of brain plasticity by direct EEG entrainment—on the other, has paved the way for a ground-breaking approach towards modifying brain function in health (Gruzelier, 2013) and disease (Birbaumer et al., 2009; Niv, 2013). Below, we revisit and elaborate on these two major themes of *control* and *plasticity* from engineering and neurobiological angles.

### Control I: an engineering perspective on neurofeedback control

Here, Arthur C. Clarke’s Third Law may prove an interesting launch pad: “Any sufficiently advanced technology is indistinguishable from magic.” At first glance, NFB could be seen as anything but “magical”, given that people universally control their brain oscillations while thinking or acting. Besides, this may be considered as NFB’s major advantage: the fact that it safely harnesses intrinsic brain processes. However, if this is merely the case, is there any reason why introducing a computer should bring anything new to the equation? Why not simply use cognitive-behavioral methods to expose and thereby modify the required brain oscillations and circuits?

**Figure 6:**
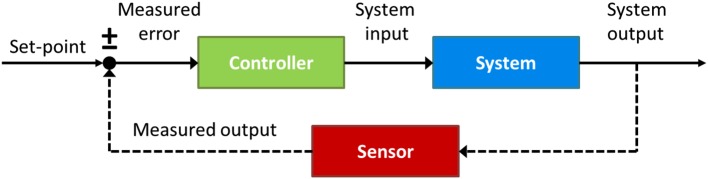
**A prototypical closed-loop control circuit**. The circuit consists of a Controller (green) which regulates the control parameter until the output of the System (blue), measured by the Sensor (red), matches the internal reference value, or set-point (±).

To answer this question it will be useful to appeal to insights from *control theory*, an interdisciplinary branch of engineering that deals with the behavior of dynamical systems with inputs, and how their behavior is modified by feedback. The cornerstone of control theory is the *feedback* loop. As depicted in Figure 6, a basic control circuit contains a *Controller* which adjusts the system’s behavior according to the real-time comparison between the output *Sensor* and the input reference value or *set-point* (±), with the goal of making this difference, or *Measured error*, zero. An illustrative example of a basic control system is the house thermostat, whereby the central heating (controller) is turned on if the current temperature (measured by the output sensor) is observed to be below the desired temperature (set-point), and keeps heating until the difference (error signal) between them is zero.

Recent research on motor control and neuroprosthestics provide convincing data that control theoretic principles can be successfully applied to model brain and behavior. At the most basic level, augmenting error-feedback proportionally improves the speed of both visuomotor (Patton et al., 2013) and BCI (Grychtol et al., 2010) adaptive learning. Remarkably, predictions from advanced models based on optimal control can match experimental data at both the behavioral (Todorov, 2004; Nagengast et al., 2009) and the neural level (Héliot et al., 2010). There is moreover a striking similarity between control system elements (controller, sensor, and set-point) and those of NFB (brain, electrodes, and reward threshold), respectively. Bearing this correspondence in mind, we can try to revisit our former question. We posit that there are (at least) two main advantages for using a closed-loop BCI to control brain activity over simple cognition or behavior. The first is based on the fact that if control is defined in its technical sense of maintaining some variable near a specified value despite disturbances, then a control system does not essentially control what it *does*. Rather, it may only successfully control the parameters that are *observable* to it i.e., what it *senses*. Hence, a thermostat performs best only when it is able to measure (observe) the temperature directly, regardless of complex heat fluctuations occurring inside or outside the house. Conversely, a thermostat without sensory access to the actual parameter of temperature, and irrespective of how complicated its internal model(s) of the environment may be, would quickly accumulate errors and eventually bring about a very large temperature drift. Given evidence that the brain respects control theoretic principles (Todorov, 2004; Marken, 2009; Grychtol et al., 2010), it is reasonable to hypothesize that the *direct sensing* accomplished by a BCI enables control of specific brain oscillations that might otherwise fall outside the scope of conscious awareness. Therefore, the first advantage of NFB may be to quite literally enlarge the cerebral *sensorium*, and thereby enable implicit control of covert brain activity that may have no direct behavioral correlate(s), e.g., activity associated with auditory hallucinations (McCarthy-Jones, 2012).

A second prospective benefit of NFB may be gleaned by considering a car’s cruise-control system, which aims to keep a car at a constant speed despite external perturbations (e.g., winds, road gradients). The system is analogous to the thermostat’s, once we exchange temperature with speed, with an important difference: the cruise control also has higher *temporal* sampling. Interestingly, feedback-control can be readily applied to the purposeful behavior of both computer (cruise control system) and human (driver), even though the physical make-up of the two systems is quite different—electrical wires, sensors, and motors in the former, but nerves, eyes, and muscles in the latter. Both the cruise control system and human driver can control only what they are able to sense or perceive to be the speed of the vehicle, respectively, albeit the human controller is far less effective at keeping the speed constant. Hence, by analogy, what can be gained by forming a human-computer hybrid for control of brain activity? Based on control-theory, we hypothesize that such a hybrid (i.e., BCI) may enable human controllers to “outsource” their own sensory-feedback processing and augment it with that of the computer, capitalizing on both its superior sensing *accuracy* and/or *temporal resolution*. A testable hypothesis that stems from this interpretation is that NFB-assisted control could prove more effective compared to an unassisted human operator. There is evidence consistent with this account indicating that NFB-regulation induces more pronounced attention (Beatty et al., 1974) and motor-cortical activation (Bai et al., 2014) than unregulated mental practice. This view is strengthened further by reports that fMRI-NFB, which has a temporal resolution on the order of seconds but a high spatial resolution, significantly boosts whole-brain signal to noise compared to covert behavior alone (Papageorgiou et al., 2013). Conversely, the lack of spatiotemporal specificity is expected to have a negative impact on NFB control, as excessively slow or spatially-distributed feedback signals may lead to an unwelcome “mixing” of irrelevant activities (Bazanova and Aftanas, 2010). Here, the specificity of NFB control could be tested on both the spatial and temporal dimensions of feedback signals, which might include brain regions predefined via inverse-source localization (Congedo et al., 2004) or rhythms that need to be controlled for a particular temporal duration/dynamic (Congedo et al., 2004; Hoedlmoser et al., 2008). In this regard, future NFB studies could also take inspiration from recent BCI approaches which have exploited machine-learning methods (Lotte et al., 2007) for identifying the individual-specific EEG patterns for training, and that may be based on *a priori* behavioral performance (Xiong et al., 2014).

The present framework implies that theoretically any observable measure of brain activity can be extracted and tested for volitional control. But what exactly constitutes successful *control*, and how best to quantify it? Generally, a strict definition of control can be formulated in the engineering sense of enhancing the *signal-to-noise* ratio of a parameter relative to a control condition (e.g., resting-state, sham, or sensory stimulation without control), which could be administered sequentially or interspersed randomly in the experiment. Hypothesis testing may then be used to test whether, during NFB in comparison with control trial(s), there is a significant difference in the mean together with a reduction (or no change) in the variability of the controlled signal. With respect to existing methods in the literature, this approach is technically equivalent to an analysis of variance (ANOVA) or a Student’s *t*-test, which similarly account for a variable’s mean and variance. If multiple confounding variables are involved, it might then be appropriate to use a multivariate analysis of covariance (MANCOVA). To date, some of the oscillatory parameters reported to be volitionally controlled include amplitude (Kamiya, 2011), frequency (Angelakis et al., 2007), phase-locking (Brunner et al., 2006) and complexity (Wang et al., 2011b). It remains to be seen in future studies to what extent new measures of brain dynamics can be harnessed, such as integration or segregation of multiple brain networks, etc.

### Control II: neurobehavioral conditioning

There is the outstanding issue of the theoretical relationship between closed-loop and “behaviorist” operant conditioning models used to describe NFB learning? “Open-loop” models assume causation runs in a one-way path from environmental input to behavioral output; the system’s output does not “loop back” and affect its input (Marken, 2009). Hence, the flow of causality is linear in the open but circular in the closed loop. According to behaviorist Stimulus-Response (S-R) theory, environmental stimuli (S) cause behavioral responses (R) via the organism, which is treated as a “black box” in between. Put simply, behaviorist perspectives see inputs causing outputs, whereas feedback implies that outputs cause inputs. The open-loop behaviorist model can technically account for classical conditioning paradigms where stimuli “cause” reflexive behavior (e.g., bell rings, dog salivates), but less convincingly explain operant behavior, which is when behavioral output (the controlled variable) is used to “cause” sensory variables (e.g., pigeon pecks, gets more food). Of course, since the closed-loop is circular, then it could appear that input causes output (more food leads to more pecks). Hence the behaviorist interpretation. However, let us consider how was the relationship established *a priori*? Inherent in any definition of causality is the notion that the effect cannot temporally precede the cause. If this is the case, during the establishment of operant conditioning, the stimulus (S) is presented *after* the correctly generated behavior (R), therefore it cannot be defined as its cause. Recent work points to an intrinsic (neural) source of behavioral variability that may underlie an animal’s attempts to “find” the appropriate behavior (Heisenberg et al., 2001). As a result, we propose that NFB learning, whether it be continuous or intermittent, may be better conceptually formulated by control-theoretic closed-loop models (Todorov, 2004; Marken, 2009; Grychtol et al., 2010). In practice, this can be condensed to the following sequence of events: initially the fluctuating feedback signal reflects stochastic (i.e., unconditioned) neural variability (Legenstein et al., 2010), consequently on random occasions this neural variability will infrequently generate activity that will meet the threshold for reward (i.e., which represents zero feedback-error); upon presentation of the sensory cue/reward, the brain may then “memorize” the distinct *neural/behavioral* state as an internal set-point, by releasing a reward-modulated signal for synaptic plasticity, e.g., dopamine (Legenstein et al., 2008). Crucially, the latter is the starting point for subsequent loops during which the human controller (with implicit/explicit neurocognitive strategies) attempts to reproduce, in a feed-forward way, the neural/behavioral state of the previously established set-point (Basso and Olivetti Belardinelli, 2006). Naturally, multiple loops (i.e., conditioning trials) will result in further refinement of the set-point, and translate to a more efficient open-loop strategy. Accordingly, recent data suggest that open-loops operate in the brain (Basso and Olivetti Belardinelli, 2006), coupled with the fact that feed-forward internal representations of input-output transformations seem to occur during motor control, so as to simulate predictions when feedback is not rapid enough (Wolpert et al., 1995). Compatible with our model, latest findings indicate that the initial stage of BCI learning is associated with activations in prefrontal, premotor, as well as parietal cortex (Wander et al., 2013), and that plasticity of cortico-striatal circuits is necessary (Koralek et al., 2012). A pertinent observation is that when NFB is given to patients with frontal lobe lesions, self-regulation of cortical activity is only successful with feedback but abolished during behavioral transfer (no-feedback) (Lutzenberger et al., 1980).

Lastly, we want to point to a likely connection between NFB and more complex neuroprosthetic learning. Although control-theoretic principles are useful for forming a conceptual understanding, the underlying “neural network” reality of learning to move a neuroprosthesis is more complex, since the number of control dimensions and signals is much higher (Perge et al., 2014). Nevertheless, this type of learning is still understood to occur through a combination of intrinsic neural variability, sensory-feedback, error-minimization, and a global reward-signal (Jarosiewicz et al., 2008; Legenstein et al., 2008, 2010).

### Control III: must neurofeedback signals be conscious? A global workspace hypothesis

Biofeedback is marked by a strikingly large range of physiological phenomena that can come under voluntary control, which apart from brain oscillations, includes autonomic functions (Cowan et al., 1990), single motor units (Fetz, 1969) and non-sensory cortical neurons (Cerf et al., 2010). In actual practice, the sensory feedback signals used in NFB are always reportable as conscious. Feedback signals are rarely if ever presented below sensory threshold, or in the presence of distractions or masking noise. Instructions generally draw the subject’s attention to the feedback signal before training. Thus intuitively we seem to assume that effective sensory feedback must involve clearly conscious stimuli. In contrast, the physiological events to be trained by NFB, like alpha activity, are generally not conscious. Neurofeedback therefore trains voluntary control over an unconscious physiological process, using conscious feedback signals. In human cognition, it is striking how few operations are conducted in a fully conscious fashion, and how much is allocated to highly practiced unconscious automatisms. Language is a well-studied example, in which only one or two “chunks” (like words or syllables), may be conscious at any moment in time, while fast and complex syntactic, semantic, word retrieval and interpersonal processes remain largely unconscious. Human beings do not consciously decompose sentences into subjects, verbs and objects; rather, in childhood we learn to perform such grammatical operations implicitly and automatically. While conscious cues may trigger syntactic operations, syntax generally operates as a large set of independent modules. Many highly practiced automatisms in the brain seem to operate in such a fashion. One major advantage of this task allocation is that automatic modules do not load central limited capacity.

Over the last 20 years, a growing experimental literature has compared physically identical stimuli that differ only in that one stimulus is conscious and reportable, and the other is not. Conscious sensory input has been shown to trigger more widespread, more coherent, and more stimulus-specific brain activity than closely matched unconscious input (Doesburg et al., 2009b; Panagiotaropoulos et al., 2012; Dehaene, 2014). Binocular rivalry is the classical example, but other techniques have been studied, including visual backward masking, selective attention, change blindness and the attentional blink. It has long been observed that cortical event-related potentials show brain-wide waveforms triggered by conscious stimuli. Baars (1988) and Baars et al. (2013) present a large body of evidence showing that conscious stimuli are widely distributed in the brain. This approach has been called Global Workspace Theory (GWT), and it has been widely tested empirically. Global “broadcasting” in the brain makes sense if we think of the brain as a massively distributed “society” of active and highly specialized neural circuits which retain local processing initiative. Such “agent societies” have been widely studied in computer science and have many biological analogs. A simple example is a college classroom in which all students are equipped with feedback clickers, allowing them to raise questions and pace the presentation rate of powerpoint slides. The speaker’s voice is distributed globally to all listeners, who make local decisions whether or not to push a feedback clicker asking the speaker to repeat or explain some point more fully. This non-hierarchical style of functioning works well in many applications.

One can think of NFB as a retrieval problem, a task of finding which particular physiological event is to be linked to the feedback signal. We may draw an analogy with trying to locate a child lost in a large city. It makes sense initially to search for the lost child around home or school, in a local and systematic fashion. But if the child cannot be found, it may help to broadcast a message to all the inhabitants of the city (e.g., via TV), to which only those who recognize it as personally relevant would respond. The message is global, but only the appropriate local units respond to it. Baars (1988) has suggested therefore that NFB may work on a very wide range of neural activities because the signal triggered by conscious stimuli is also distributed very widely in the nervous system. If local alpha sources can generate alpha oscillations, for example, their routine operations may not require conscious involvement or voluntary control. In the special case in which alpha activity evokes conscious feedback, alpha sources may come under voluntary control of the feedback signal (Kamiya, 2011). This is only possible if the feedback signal is widely distributed, as conscious stimuli appear to be. An easily testable prediction follows from these points, namely that a visual feedback signal that is not conscious due to backward masking or binocular rivalry would not work to establish feedback control, even if it were physically identical to the conscious input.

Recently it was shown with intracranial recording in epileptics that NFB permits patients to control single-neuron firing in the temporal lobe (Cerf et al., 2010). Similar findings have been reported in animals (Fetz, 1969). This finding suggests another testable prediction: in epileptic patients who are medically required to wear an implanted cortical electrode grid before brain surgery, a single electrode could be randomly selected among a typical 64-lead grid. If epileptic patients can learn to arbitrarily select any one of 64 electrodes on cue, via conscious feedback, one could measure the patient’s accuracy against the *a priori* random probability of controlling 1 out of 64 electrodes at a specific time. This would yield a quantitative index of transmission accuracy from the response-contingent conscious feedback signal to the selected recording electrode. These data could also be analyzed using signal detection theory (i.e., receiver-operating characteristic), mutual information (a measure of neural transmission volume), Tononi’s phi (Tononi, 2004), and the like.

### Plasticity I: Hebbian mechanisms of plasticity

The last decade has witnessed a surge of interest in the topic of brain plasticity and the genuine promise it holds for fostering brain health and reversing pathology (Ganguly and Poo, 2013). Although many different techniques can be used to manipulate neural plasticity, either through sensory, pharmacological, optogenetic or electromagnetic interventions, these approaches may fall short when it comes to answering how the intact brain is able to regulate its plasticity intrinsically, i.e., independently of any external stimulus or substance. Studies have indeed reported correlational evidence for intrinsic plasticity, (Tsukamoto-Yasui et al., 2007), yet animal experiments of this kind are prohibitive in humans. An elegant way this question can be causally approached in humans is via NFB, given that it permits identical sensory stimuli and equivalent frequencies of reward to be used across all users, effectively clamping the external milieu. Hence, participants’ entrained neuronal (M/EEG) differences may be considered as resulting minimally from external factors and can instead be regarded as being driven by the modulation of intrinsic, stimulus-independent brain states (Poulet and Petersen, 2008; Zagha and McCormick, 2014). This makes NFB a unique tool for establishing a causal link between endogenous brain oscillations and their cognitive-behavioral functions.

Akin to general learning processes such as skill or language acquisition, NFB usually requires repeated applications of individual “training” sessions of about 20–60 min each, occurring on separate days and spread out over weeks or months depending on the person’s response. Accumulating data suggest that maintaining the cortex in a persistent oscillatory pattern via NFB effectively “conditions” the neuronal circuits to produce the same pattern with a higher probability in the future (Sterman et al., 1970; Lubar and Swartwood, 1995; Cho et al., 2008; Ros et al., 2010). At present, the molecular substrates underpinning this *long-term* training effect still remain to be elucidated. However, they may be theoretically explained by evidence that the magnitude of an EEG oscillation increases with the number of neurons/synapses giving rise to it (Musall et al., 2012), combined with the proverbial Hebbian principle that “synapses that fire together wire together, and synapses that fire apart wire apart” (Knoblauch et al., 2012). Consequently, during amplified or “synchronized” oscillations, the population(s) of neurons which are coherently involved in generating an oscillatory pattern would, after some time, further strengthen the connections between themselves, thus making it easier for this population pattern to emerge once again in the future. Conversely, maintaining a group of neurons in a prolonged desynchronized state would weaken the correlated firing of their synapses and attenuate the connections that give rise to synchronization. These outcomes have recently been mathematically modeled *in silico* with neural network models of spike-timing dependent (STDP) Hebbian plasticity (Pfister and Tass, 2010; Zaehle et al., 2010; Knoblauch et al., 2012) and respectively validated *in vivo* by *synchronizing* transcranial alternating current stimulation (tACS; Zaehle et al., 2010) and *desynchronizing* electrostimulation of hippocampal circuits (Tass et al., 2009). In accordance with this model, high-frequency (>90 Hz) DBS can successfully supress low-frequency oscillations (~9 Hz) in Parkinson’s disease, leading to an improvement of symptoms, while low-frequency (<50 Hz) DBS can exacerbate them (McConnell et al., 2012). Importantly, symptom reduction is further improved when stimulation is performed in a closed-loop, and matched to the frequency of the abnormal oscillations (Rosin et al., 2011).

**Figure 7:**
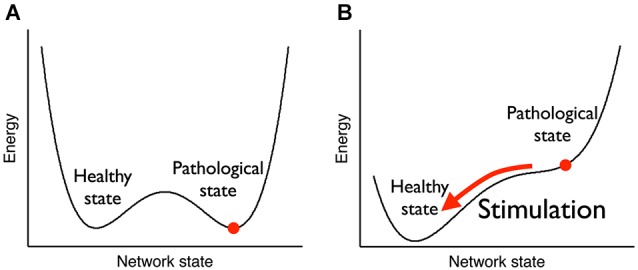
**Attractor landscape pre-post therapeutic stimulation**. **(A)** Before stimulation, both the pathological state (strong weights, high neuronal synchronization) and the healthy state (weak weights, low neuronal synchronization) are stable, i.e., they are local minima of an abstract energy function. **(B)** During stimulation, the pathological state becomes unstable and the network is driven towards the healthy state. After stimulation has stopped, the network stays in the healthy state. From Pfister and Tass (2010).

**Figure 8:**
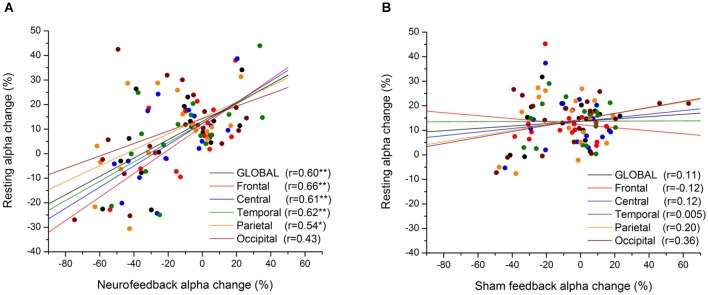
**Short-term Hebbian plasticity following neurofeedback (NFB)**. Scatter-plot of mean alpha amplitude change across electrodes during feedback vs. resting state (post-feedback), for NFB **(A)** and SHAM **(B)** groups. The anatomical location of each subgroup of electrodes is represented by a different color (see legend). *p < 0.05, **p < 0.01. From Ros et al. (2013).

Likewise, coordinated sensory (acoustic) stimulation seems a promising approach for treatment of tinnitus, revealing long-term reductions in slow-frequency rhythms (Adamchic et al., 2014). Hence, as illustrated in Figure 7, mechanisms of neural desynchronization can be harnessed to reverse over-pronounced (pathological) oscillations which have formed due to excessive synaptic connectivity, by tuning the network into a less-synchronized basin of attraction (Pfister and Tass, 2010). In light of these empirical and modeling results, it is reasonable to expect that similar Hebbian plasticity mechanisms are likely to be at work during endogenous entrainment (synchronization) or extinction (desynchronization) of EEG rhythms with NFB training (Legenstein et al., 2008). Here we select one representative example of the former and latter from the already abundant literature, revealing short-term (<1 day) and long-term (>1 day) changes in rhythmogenesis. To begin with, Sterman et al. (1970) were the first to show that brain oscillations operantly conditioned in awake cats augmented the same type activity during subsequent sleep (<1 day), and even 1 month after termination of training (>1 day). Recently, Cho et al. (2008) have reported a positive correlation (*r* = 0.7) between alpha oscillation amplitude at the end of a NFB session and the following session’s resting-state (>1 day). As shown in Figure 8, the same positive relationship (*r* = 0.6) is observed between oscillatory power during NFB and the immediate post-session resting-state (<1 day), but this time for alpha-*desynchronizing* (supressing) NFB, controlled by a sham-feedback group (Ros et al., 2013).

**Figure 9:**
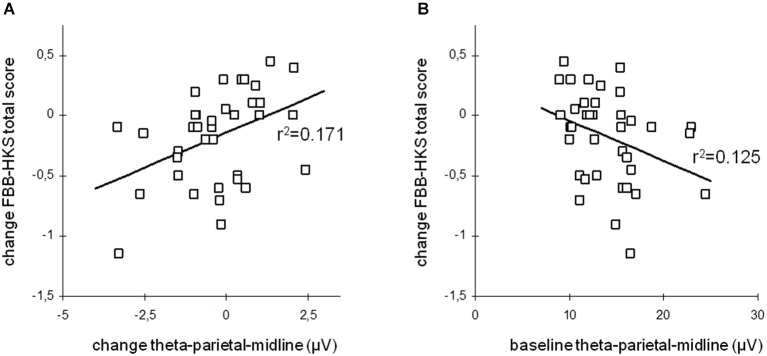
**Long-term Hebbian plasticity following neurofeedback (NFB)**. Theta oscillation amplitude vs. ADHD clinical score change. **(A)** Change of total ADHD score vs. post-NFB change of theta activity. **(B)** Change of total ADHD score vs. pre-NFB theta activity (baseline). From Gevensleben et al. (2009).

This change in resting-state desynchronization was observed to induce a temporally-direct increase of cortical excitability and disinhibition probed via transcranial magnetic stimulation (TMS; Ros et al., 2010), suggesting a causal link between NFB entrainment and changes in intrinsic brain state (Poulet and Petersen, 2008). Moreover, this finding highlights the ability of NFB to impact the excitation/inhibition balance of cortical circuits, thereby potentially tuning system criticality (Poil et al., 2012). An interesting neurobehavioral consequence of alpha desynchronizing NFB is that it enhances functional connectivity within a large-scale resting-state network implicated in intrinsic alertness (“salience network”), correlating with decreased reaction time and frequency of mind-wandering (Ros et al., 2013). Consistent with a circular causality between mind and brain (Freeman, 1999), NFB is thus able to simultaneously impact brain dynamics, mental phenomena and behavior, justifying its promise as a next-generation treatment for neurological and psychiatric disorders. For this reason, we refer to a NFB randomized controlled trial that neatly demonstrates the linkage between clinical improvement and modulations of intrinsic EEG activity in children with ADHD (Gevensleben et al., 2009). The effects are detailed in Figure 9A below, disclosing a positive relationship between changes in resting-state EEG synchronization and changes in overall ADHD symptoms (FBB-HKS score), i.e., the children showing greatest attenuations of their theta amplitude (consistent with the NFB protocol), exhibited the largest improvements in clinical scores. Interestingly, as shown in Figure 9B, these improvements were furthermore predicted by pre-training (baseline) levels of synchronization, where children presenting the most pronounced theta amplitudes at intake had largest benefits from the NFB training. This outcome is entirely consistent with findings implicating theta excess as a candidate biomarker of ADHD (Chabot et al., 2005; Snyder et al., 2008).

### Plasticity II: homeostatic plasticity

Despite the appealing correlations presented in the earlier section, they seem to tell only one side of the story. It so happens that *intra*-individual variation in brain plasticity induction appears to be equally, if not more, pronounced than *inter*-individual differences. A review of recent studies with non-invasive brain stimulation reports evidence of what is referred to as homeostatic plasticity or “metaplasticity” (Abraham, 2008; Ridding and Ziemann, 2010). Essentially, even though group effects are proven to be reliable, they generally mask a large amount of intra-individual variability from test-to-retest, i.e., variable excitability changes on different days (Fratello et al., 2006). Here, the history of prior learning (plasticity induction) in the brain inversely determines the degree of subsequent plastic changes, by following the so-called Bienenstock-Cooper-Monroe (BCM) rule (Cooper and Bear, 2012). In simpler terms, prior increases in synaptic strength (e.g., LTP-like) are more likely to be accompanied by decreases in synaptic strength (e.g., LTD-like) later on if the same induction paradigm is repeated (Müller-Dahlhaus et al., 2008), and vice versa. The brain, it seems, continuously oscillates between well-defined extremes of high and low synaptic strength (Tononi and Cirelli, 2006). This appears to be the consequence of physiological and computational ceiling pressures which occur naturally in synapses, the molecular mechanism of which is still under investigation (Abraham, 2008). Homeostatic plasticity may aid in our understanding why NFB also produces variable intra- and inter-individual effects. Hence, oftentimes changes in EEG synchronization occur in very opposite direction as would be expected according to Hebbian plasticity.

**Figure 10:**
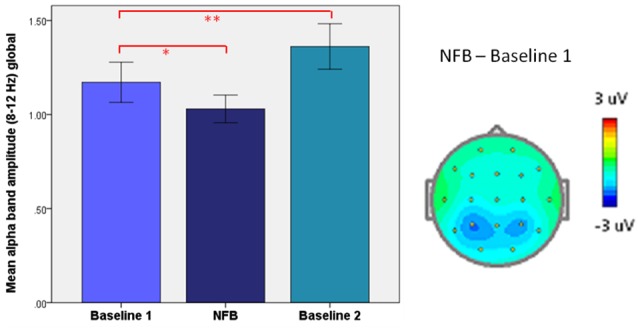
**Homeostatic “rebound” following desynchronizing neurofeedback (NFB)**. **Left**: mean (±SEM) global alpha amplitude in PTSD patients: before (Baseline 1), during (Neurofeedback), and right after neurofeedback (Baseline 2). **Right**: Topographic plot of mean alpha amplitude change during neurofeedback (NFB), relative to resting-state (Baseline 1). *p < 0.05, ** *P* < 0.005. From Kluetsch et al. (2014).

As depicted in Figure 10, we have previously reported on a paradoxical “rebound” of EEG synchronization immediately following alpha-desynchronizing NFB in patients with PTSD, which related to increases in subjective well-being (Kluetsch et al., 2014). Here, alpha synchronization during NFB *negatively* correlated with post-NF resting state changes. The important aspect to note here is that PTSD patients have abnormally reduced alpha-power at baseline (i.e., in the resting state) (Jokić-Begić and Begić, 2003). Hence, this may in effect be quite a logical outcome, since who could expect the Hebbian form of plasticity to perpetuate *ad infinitum*, leading to pathologically excessive or reduced oscillations and compromising their essential function? Evidently, as phenomena of epileptic hypersynchrony and flat-line coma suggest, there is good reason why the brain keeps its oscillations in check.

A related phenomenon is the spectral over-synchronization frequently seen following mental fatigue (Huang et al., 2008) or sleep deprivation (Gorgoni et al., 2014), understood to be the product of increases in local experience-dependent plasticity (Hung et al., 2013). Subsequently, following sleep, the EEG is miraculously restored to a less synchronized state the day after (Plante et al., 2013). Fascinatingly, this latter process seems to be compromised in psychiatric disorder (Plante et al., 2013). Tying all this evidence together appears to lead to a beautifully parsimonious conclusion: it is neither high nor low synchronization that may be critical, but rather a golden balance in-between. In addition to abnormalities reported in clinical populations (Coburn et al., 2006), some investigations directly consistent with this view observed that intermediate levels of synchronization best predict conscious perception (Linkenkaer-Hansen et al., 2004), whilst both high and low spectral power are associated with attentional impairment (Pezze et al., 2014). Interestingly, the latter appears to be due to oppositely extreme shifts in the excitatory/inhibitory balance of the prefrontal cortex (Pezze et al., 2014). As discussed in the previous chapter, pathological oscillations can manifest themselves as either low or high synchronization extremes when compared to normative populations. This can equally apply to long-range phase synchronization (e.g., increased phase-locking of alpha rhythm in cognitive impairment, López et al., 2014) as to locally-generated oscillation amplitude (e.g., over-pronounced beta power in Parkinson’s, Little and Brown, 2014).

In our view, many brain pathologies could thus be succinctly characterized as disorders of homeostatic plasticity, in light of the above evidence as well as the fundamental links between brain oscillations and synaptic potentiation (Tsukamoto-Yasui et al., 2007; Vyazovskiy et al., 2008; Tsanov and Manahan-Vaughan, 2009). This could be especially the case for non-degenerative brain disorders (e.g., ADHD, epilepsy, PTSD etc.), where functional abnormalities are likely not associated with progressive cell loss. In other words, non-degenerative brain disorders may have a *self-tuning impairment*, having lost their dynamic repertoire by being “trapped” in an abnormal resting-state oscillatory pattern (Ghosh et al., 2008). If this is correct, then one might expect measures of neural variability to be lower in brain disorders during task-free conditions. Several reports appear to support this hypothesis, as fluctuations of EEG synchronization are indeed diminished in brain disorders, including Alzheimer’s (Stam et al., 2005), psychosis (Müller et al., 1986), OCD (Drake et al., 1996), tinnitus (Schlee et al., 2014) and ADHD (Woltering et al., 2012). During task conditions, however, the relationship can be more complicated seeing that a decrease in variability would indicate more stable “locking” into a particular brain state, which may or may not facilitate task performance (Stam et al., 2002; Deco and Hugues, 2012). An excellent example of this is how stronger theta, but weaker alpha synchronization variability is associated with better performance during a working memory task (Stam et al., 2002). Yet, given evidence of a common functional architecture between resting and task conditions (Smith et al., 2009; Krienen et al., 2014), it is reasonable to posit that the more variable dynamic range of tonic (i.e., resting-state) EEG may underpin that of the phasic (i.e., task-related) EEG, characterized by so called event-related oscillations (EROs), which have been strongly implicated in cognition (Klimesch et al., 2001; Neuper and Pfurtscheller, 2001). Hence, in light of the aforementioned physiological ceiling effects, it is plausible that resting-state hyper-and hypo-synchrony may dimensionally-restrict the dynamic range of phasic event-related synchronization (ERS) and event-related desynchronization (ERD) patterns (Yordanova and Kolev, 1998; Wascher et al., 2014), respectively. To be exact, we speculate that the *relative* amount of ERS (ERD), represented by percent signal change from baseline (spontaneous) activity, could be reduced in disorders presenting hyper (hypo) synchronization. Neurofeedback designs could thus be made to target either tonic or phasic EEG, given this inextricable linkage between them.

Lastly, we would like to outline two types of homeostatic plasticity, by defining *elastic* homeostatic adaptation as adaptation that does not cause any persistent changes in the system, and *plastic* homeostatic adaptation as adaptation where there is a persistent change in some part of the system (Williams, 2006). The elastic form may be related to short-term changes (<1 day) in EEG synchronization, such as the wake-sleep cycle, or even ERO dynamics themselves (Neuper and Pfurtscheller, 2001). A good example of an elastic homeostatic adaptation after NFB might be the rebound observed by Kluetsch et al. (2014). However, especially relevant to therapeutic applications of NFB may be plastic homeostatic adaptation (>1 day), whereby the homeostatic set-point of the system may be tuned *lastingly*. Here again we revisit control theory, by envisioning a plastic re-tuning of resting-state oscillations towards a new mean (set-point); precisely what is intended by, and classically observed after, NFB therapy (Lubar et al., 1995; Gevensleben et al., 2009). However, the main reason why this mechanism should be considered homeostatic, rather than simply plastic, is in order to also accommodate observations of long-term rebound phenomena. An interesting example supporting this model is a recent NFB study demonstrating a *long-term* (>1 day) alpha rebound in children with ADHD (Escolano et al., 2014), despite evidence of alpha desynchronization within training sessions. This account is further strengthened by reports that bidirectional (up/down) NFB training normalizes targeted ADHD band-powers toward group mean values (Liechti et al., 2012). Hence, as a consequence of homeostatic plasticity, a key prediction of the proposed framework is that both unidirectional and rebound NFB outcomes may be permissive toward normalizing pathological brain oscillation measures (e.g., power, phase-locking, peak frequency, 1/f), as well as the dynamical landscape that subserves them. From this perspective, NFB training could be seen to “tune” the brain’s intrinsic mechanisms of homeostasis, which are used to self-organize towards an optimal (i.e., near-critical) set-point following a period of adaptive plasticity (Hsu and Beggs, 2006), but which have become maladaptive in pathology.

### Plasticity III: structural plasticity

Thus far, we have concentrated on aspects of functional brain activity, yet it is now firmly established that there is an inseparable connection between brain structure and brain function (e.g., Pizoli et al., 2011). Although the brain has often been compared to the functioning of a computer, it differs from the former in a crucial respect: in a traditional computer the physical architecture (i.e., hardware) running the program is not modified by the computations (i.e., software). Instead, in the brain the physical connection strengths making up the neural networks are shaped by their intrinsic activity (i.e., it is a form of “wetware”). On the one hand, the structural pathways in the brain undergrid the flow of neural activity, much like roads shape the flow of traffic (Haimovici et al., 2013). Unsurprisingly then, white-matter integrity has been associated with parameters such as the alpha peak frequency (Valdés-Hernández et al., 2010), while gray-matter is found to positively correlate with EEG power during brain maturation (Whitford et al., 2007). Consistent with this, NFB control of brain oscillations can be predicted by the morphology of underlying cortical generators (Enriquez-Geppert et al., 2013) or associated white-matter pathways (Halder et al., 2013). On the other hand, traffic (brain) dynamics is an emergent process which is governed by the behavior of the drivers (neural activities), e.g., traffic jams may result from a temporal upsurge of activity. Subsequently in the brain, akin to strategic road construction, pathways become reinforced or weakened in response to neural activities through a process known as activity-dependent plasticity (Butz et al., 2009; Ganguly and Poo, 2013). Such “remodeling” involves receptor trafficking, myelination plus spine formation (Butz et al., 2009) and may occur at different timescales, from less than 1 h (Munz et al., 2014) to days (Butz et al., 2009). This symbiotic interplay between structure and function, which defines self-organizing systems, is at the heart of NFB’s therapeutic potential: by targeting dynamic activity alone one can unlock and induce changes in the brain’s structural architecture, which would in turn support a more persistent functional reorganization. After 50 years since NFB’s inception, a recent study has finally provided empirical support for this effect, reporting gray and white-matter increases following a total of 20 h of training in healthy subjects (Ghaziri et al., 2013). If NFB is truly able to “hard-wire” the brain, then one should expect a certain stability of effects *post* intervention. This is indeed observed to be the case: behavioral improvements are robustly conserved at long term follow-up in ADHD (6 months, Gevensleben et al., 2010; Steiner et al., 2014), autism (12 months, Kouijzer et al., 2009), alcoholism (18 months, Watson et al., 1978), learning-disability (2 years, Becerra et al., 2006), and epilepsy (10 years, Strehl et al., 2014). Crucially, in the only study of its kind to date, positive behavioral changes were associated with a sustained, maturational improvement of the resting-state EEG (Becerra et al., 2006).

Let us return to the traffic analogy for a final reflection: the topology (i.e., spatial organization) of road networks is not random but contains a small proportion of long-range highways and a greater proportion of more clustered, local roads. Remarkably, both road networks and brain networks have been observed to exhibit this principle of organization, obeying what has been termed a “small-world” structure. In light of physical constraints and wiring costs, there appears to be an optimal balance between distributed and local connectivity that affords efficient network performance (for a review see Bullmore and Sporns, 2009). However, perhaps the most striking revelation is that a small-world topology apparently facilitates systems to achieve criticality (Russo et al., 2014) and self-generate oscillations (Wang et al., 2011a). We thus seem to have come full circle: the development of a healthy brain requires that it homeostatically organizes both functionally (Boersma et al., 2011) and structurally (Butz et al., 2014) towards a small-world architecture. If this is true, functional abnormalities due to pathological oscillations would firstly be suggestive of an anomalous topological structure (consistent with Stam, 2014), but, moreover, that normalizing them via NFB would re-establish a small-world network organization. At present, the latter is an intriguing hypothesis that remains to be tested.

This ultimately leads us to the topic of unspecific changes and some evident caveats, given that NFB has been known to induce unpredictable effects on local as well as distributed EEG signatures. For example, long-term training to raise theta (4–8 Hz) over alpha (8–12 Hz) power at parietal sites was associated with a post-training reduction of faster beta (14–18 Hz) activity in the prefrontal cortex (Egner et al., 2004). Initially, this outcome could be explained by an overall leftward shift in central frequency due to entrainment of lower-frequency rhythms. However, it should be borne in mind that intact brain reorganization is assumed to be regulated via complex homeostatic interactions (Butz et al., 2009). As we have argued above, plastic changes cannot necessarily be expected to follow a linear path when the underlying topology is strongly non-linear (e.g., small-world). Moreover this conundrum inevitably holds true for all interventions, extrinsic or intrinsic, which deal with the brain and its panoply of networks (Mangia et al., 2014). Nevertheless, we believe this is all the more reason to explore the brain’s innate capacity for self-organization: the sooner its mechanisms are elucidated, the better will be our prospects to exploit them.

## Closing remarks: why neurofeedback?

Apart from some interesting insights on how the brain’s resident orchestra may tune its rhythms, we would be remiss not to discuss whether NFB might possess any real therapeutic advantage(s) over currently available techniques? Most of them, including pharmacotherapy and non-invasive brain stimulation (rTMS, tDCS), are also known to modulate brain oscillations, albeit indirectly. So one should technically ask, why NFB? We contend that NFB’s chief strength may not only rest in its direct control of brain oscillations, but in its *safety* and *long-term stability*. When applied judiciously, reported adverse effects of NFB are very rare (Hammond, 2010), and most appear limited to mild headaches which resolve in the aftermath of training. In comparison to the well-known side effects of medications and the exceptional but grave complications that may ensue from electromagnetic stimulation (Rosa et al., 2006), NFB could be regarded as the more favorable option safety-wise. Furthermore, being artificial, transcranial stimulation techniques produce electromagnetic driving forces that are not intrinsic to the brain, and thus still need to be validated for their long-term safety (Davis, 2014). Therefore, the fact that NFB may produce changes under physiologically-normal conditions may be its greatest asset. Interestingly, this very property may be responsible for another, arguably even more fundamental benefit: long-term stability. A distinguishing feature of NFB is that it is purely endogenous, whereby self-organization is invoked by the system itself, i.e., from the “inside out” rather than from the “outside in”. This could ultimately minimize treatment tolerance/withdrawal and prove to be a critical distinction, given collective evidence that the brain obeys principles of homeostasis, combined with reports of NFB’s exceptionally persistent effects (e.g., Strehl et al., 2014). In light of the amazing plasticity displayed by the human brain, the prospect that such an approach could offer is important and urgent enough to motivate future investigations so as to further validate the extent of its impact on normal and pathological brain function. The fruits of such an inquiry could lead to a remarkably safe, non-invasive and above all natural approach for directing neuroplastic change.

## Conflict of interest statement

The authors declare that the research was conducted in the absence of any commercial or financial relationships that could be construed as a potential conflict of interest.
